# Knowledge of Prevention of Mother‐to‐Child Transmission of HIV and Associated Factors Among Pregnant Women in Debre Birhan, Ethiopia: A Community‐Based Cross‐Sectional Study

**DOI:** 10.1002/hsr2.73164

**Published:** 2026-09-01

**Authors:** Lewegneh Wegayehu Tessema, Yazachew Moges Chekol, Elsa Awoke Fentie, Abebaw Addis Gelagay

**Affiliations:** ^1^ Department of Midwifery Gambella Teachers Education and Health Science College Gambella Ethiopia; ^2^ Faculty of Medicine, Dentistry and Health Science University of Melbourne Melbourne Australia; ^3^ Department of Reproductive Health, College of Medicine and Health Science, Institute of Public Health University of Gondar Gondar Ethiopia

**Keywords:** human immunodeficiency virus, knowledge, pregnant, women

## Abstract

**Background and Aims:**

Vertical transmission of HIV accounts for over 90% of pediatric HIV infections worldwide, making it a major public health concern. Maternal knowledge of mother‐to‐child transmission of HIV and its prevention is essential for the uptake of preventive measures and the success of elimination strategies. This study aimed to assess knowledge of prevention of mother‐to‐child transmission of HIV and associated factors among pregnant women in Debre Birhan, Ethiopia.

**Methods:**

A community‐based cross‐sectional survey was conducted from June 2 to July 8, 2022 in Debre Birhan town. Participants were selected using a simple random sampling technique. Data were collected through a structured questionnaire, entered into Epi Data version 4.6, and exported to Stata version 17 for analysis. Prevalence ratios with 95% confidence intervals were estimated using Poisson regression with robust variance to identify factors associated with knowledge of PMTCT.

**Results:**

Of 816 pregnant women, 803 provided complete information (response rate of 98%). Only 35.87% (95%CI = 32.62–39.25) of pregnant women demonstrated good knowledge of PMTCT. Factors associated with knowledge of PMTCT included residence, educational status, occupation, and receiving health information from healthcare providers.

**Conclusion:**

Only 35% of pregnant women in Debre Birhan demonstrated good knowledge of PMTCT. Knowledge was significantly associated with residence, occupation, education, and receipt of health information from providers. These findings highlight the need for targeted interventions, including strengthened behavioral change communication through health extension workers and integration of PMTCT education into routine antenatal care, to support Ethiopia's HIV prevention goals.

**Abbreviations**
AIDS: Acquired ImmunoDeficiency Syndrome
ANC: Antenatal Care
ART: Antiretroviral Therapy
HIV: Human Immuno Virus
KMO: Kaiser–Meyer–Olkin
MTCT: Mother‐to‐Child Transmission
PCA: Principal Component Analysis
PMTCT: Prevention of Mother to‐child‐Transmission
UNAIDS: Joint United Nations Program on HIV/AIDS

## 1 Background

Mother‐to‐child transmission (MTCT) of HIV refers to the transmission of the virus from an infected mother to her child during pregnancy, at the time of labor and delivery, or through breastfeeding. Each year, an estimated 1.3 million women and adolescent girls living with HIV become pregnant worldwide [1]. Without preventive interventions, the transmission of HIV from mother to her child during pregnancy, labor, delivery, or breastfeeding ranges between 15% and 45% [1]. In sub‐Saharan Africa, women and girls account for 63% of new HIV infections, and vertical transmission contributes to over 90% of pediatric HIV infections [2, 3]. In 2024, an estimated 9.2 million people worldwide needed HIV treatment but were not receiving it, and half of these individuals lived in Sub‐Saharan Africa [4]. By the end of 2024, global efforts had reduced new HIV infections by 40% and AIDS‐related deaths by 56% compared to 2010, reflecting significant progress in the fight against HIV/AIDS. However, a sudden collapse in funding created a crisis in the global AIDS response. Despite reductions in mortality, prevention gaps persisted, with 1.3 million new infections reported in 2024, nearly unchanged from the previous year [4]. These highlight both the achievements and the ongoing challenges in sustaining global HIV prevention and treatment. In 2023, UNAIDS estimated that 610,000 people were living with HIV in Ethiopia, of whom 510,000 were receiving antiretroviral therapy [5].

The global community has committed to eliminating mother‐to‐child transmission (MTCT) of HIV as a public health priority [6]. This commitment is pursued through a harmonized and integrated approach aimed at improving health outcomes for both mothers and their children. Since the introduction of PMTCT programs, an estimated 2.1 million deaths and 4.4 million HIV infections have been averted among pregnant women and children [7]. However, progress is not fast enough to reach the 2030 targets set by UNAIDS (Joint United Nations Program on HIV/AIDS) and partners as part of the UNAIDS Global Strategy to End AIDS, underscoring the need for intensified efforts to sustain and accelerate prevention measures [7]. Strengthening community‐level knowledge, service accessibility, and adherence support remains critical to closing the gap and achieving the global goal of ending AIDS as a public health threat.

Ethiopia is progressing toward the UNAIDS 95–95–95 targets, with 90% of people living with HIV aware of their status, 94% of those diagnosed receiving therapy, and 96% of treated individuals achieving viral suppression. Recent pauses in U.S. foreign assistance threaten Ethiopia's HIV response, which relies heavily on external funding; PEPFAR alone provides 53% of national HIV financing, and service disruptions place thousands in Ethiopia and millions globally at risk [5].

A study revealed that women's knowledge of PMTCT ranges from 13.9% (Comoros) to 75.4% (Zimbabwe) in sub‐Saharan countries [8]. In sub‐Saharan countries, a significant number of women living with HIV are not receiving treatment during pregnancy due to limited community knowledge [9]. Knowledge of MTCT and PMTCT for HIV/AIDS is associated with characteristics such as maternal age, education, wealth status, occupation, marital status, and media exposure [10, 11, 12, 13]. A major challenge to provide PMTCT services is women's knowledge deficits [14]. Lack of knowledge about MTCT in the community negatively impacts PMTCT uptake and consequently increases HIV prevalence among children [15]. Previous facility‐based studies recommend that further research needs to be conducted on PMTCT knowledge among pregnant women in the community level [13, 16, 17].

In Ethiopia, most studies on women's knowledge of MTCT and PMTCT have been facility‐based, focusing on women who attend antenatal care or HIV testing services. While informative, these studies are limited in representativeness and do not capture women who remain outside the formal health system. Their findings have also been inconsistent, with wide variation in reported knowledge levels and associated factors. Notably, several of these facility‐based studies explicitly recommended that future research be conducted at the community level to better understand knowledge gaps among pregnant women who may not access routine services.

To address this recommendation and fill a critical research gap, our study conducted a community‐based cross‐sectional assessment of PMTCT knowledge in Debre Birhan. The novelty of this approach lies in its ability to generate representative, context‐specific evidence that reflects the experiences of women both within and outside the health system. This study provides new insights that can inform locally tailored HIV prevention strategies and strengthen Ethiopia's progress toward the UNAIDS 95–95–95 targets. So, this study aims to assess knowledge of prevention of mother‐to‐child transmission of HIV and associated factors among pregnant women in Debre Birhan, Ethiopia.

## 2 Methods

### 2.1 Study Setting, Design, and Period

A community‐based cross‐sectional study was conducted from June 2 to July 8, 2022, in Debre Birhan Town Administration. Debre Birhan Town, the capital of the North Shewa Zone in the Amhara Region, is located 130 km from Addis Ababa and 635 km from Bahir Dar, the regional capital. The town has a total population of 108,825, of whom 59,617 are female. The estimated number of women of reproductive age (15–49 years) is 25,661, and the total number of pregnant women in the town was 2135 at the time of the study. Health infrastructure in Debre Birhan includes 14 health posts, eight health centers, one government comprehensive specialized hospital, and one private hospital. The town administration is divided into 14 kebeles, comprising nine urban and five rural kebeles

### 2.2 Study Participants

All pregnant women who lived in the Debre Birhan town administration during data collection and lived there for at least 6 months were included in this study. Pregnant women who were critically ill or unable to respond to the survey were excluded from the study.

### 2.3 Sample Size Determination and Sampling Procedure

The sample size was determined using a single population proportion formula, based on the following assumptions: knowledge of PMTCT of HIV at 22.4% [18], a margin of error of 3%, a *Z* value of 1.96 at 95% confidence interval, and a 10% allowance for non‐response. This yielded a final sample size of 816. Debre Birhan town comprises 14 kebeles. The total sample size was proportionally allocated across all kebeles according to household size to ensure representativeness. Within each kebele, households were selected using a computer‐generated simple random sampling technique. A sampling frame was developed for each kebele using family folders maintained by health extension workers, which contain updated household and demographic information. In Ethiopia, health extension workers update the list of pregnant women monthly for each kebele with the support of the women's development army. From each selected household, one pregnant woman was recruited. If more than one eligible participant was present, one was chosen at random. In cases where no eligible participant was found, the next household on the list was approached. This procedure ensured proportional distribution of the sample and minimized bias.

#### 2.3.1 Dependent Variable

Knowledge (yes/no).

#### 2.3.2 Independent Variables

##### 2.3.2.1 Socio‐Demographic Factors

Age, religion, residence, marital status, educational status, occupation, wealth index, and distance from Health facility.

##### 2.3.2.2 Reproductive‐Related Factors

Parity, gravida, gestational age, current antenatal visit, number of ANC, and ANC during last pregnancy.

##### 2.3.2.3 Information‐Related Factors

Receiving health information from health care provider, discussing with husband about MTCT and HIV, ANC discussion with husband, and media exposure.

#### 2.3.3 Operational Definitions

##### 2.3.3.1 Knowledge Assessment Tools and Measurement

Knowledge of PMTCT among pregnant women was assessed using 10 dichotomous (yes/no) items. Correct responses were scored as “yes” (1 point), while incorrect responses were scored as “no” (0 points). The total knowledge score was obtained by summing all responses, yielding possible scores from 0 to 10. The tool was adapted from a previous study and pretested among 5% of the sample prior to data collection. Internal consistency was acceptable, with a Cronbach's alpha of 0.73. Prior to categorization, the distribution of knowledge scores was examined and found to be normally distributed. In a normal distribution, the mean is the most appropriate measure of central tendency, as it reflects the central value without distortion from extreme scores. Therefore, the sample mean was used as the cutoff point, and participants scoring at or above the mean were categorized as having good knowledge, while those scoring below the mean were categorized as having poor knowledge [18, 19].

##### 2.3.3.2 Wealth Index

Thirty household data items, including ownership of selected assets, type of water access, and sanitation facilities, were used to construct the wealth index through principal component analysis (PCA). All continuous and categorical variables were dichotomized between “0” and “1,” with continuous variables categorized based on quantity. Prior to extraction, sampling adequacy was assessed using the Kaiser–Meyer–Olkin (KMO) measure, which yielded a value of 0.79, indicating suitability for factor analysis. Bartlett's test of sphericity was significant (*χ* ^2 ^= 512.4, *p* < 0.001), confirming that the correlation matrix was appropriate for PCA. Variables with communalities below 0.5 were excluded, and the analysis was repeated until all retained variables met the inclusion criteria. Components with eigenvalues greater than 1 were retained, with the first component explaining 32.6% of the total variance. Factor scores were computed using the regression‐based method to generate a single composite variable representing household wealth status. The final scores were ranked into five quintiles (first to fifth) and coded as poorest, poorer, middle, rich, and richest, respectively. This approach ensured methodological rigor and comparability with similar studies [20].

##### 2.3.3.3 Media Exposure

Media exposure is defined as when women read newspapers or listen to the radio or watch television at least once a week, so if the woman is practicing among the three channel options (watching TV, listening to the radio, reading newspapers), it is considered that the woman is media exposed in our findings [20].

##### 2.3.3.4 Average Time to Reach Health Facility

The average time to reach a health facility was measured based on the mothers' reports of walking hours to health facilities. This was coded 1 if mothers reported walking hours to reach the nearby health facility to be ≥ 30 min and 0 if it was less than 30 min [21].

### 2.4 Data Collection Tools and Quality Control

Data were collected using a structured survey instrument. The questionnaire was initially developed in English, translated into Amharic to ensure clarity and cultural appropriateness, and then back‐translated into English to maintain consistency. One day of training was provided to seven bachelor's degree midwives who served as data collectors. Pregnant women were interviewed using the pretested structured questionnaire after providing informed verbal consent. The consistency and validity of the questionnaire were assessed in a pretest involving 5% of participants with similar sociodemographic characteristics in Sholagbeya town, and necessary modifications were made based on the pretest results.

### 2.5 Data Processing and Analysis

Data were entered into Epi‐Data version 4.6 to minimize errors and incompleteness, and subsequently exported to STATA version 17 for analysis. Prior to analysis, data were cleaned using frequency checks, listings, and sorting to identify missing values, with corrections made by revisiting the original questionnaires. Descriptive statistics were summarized using frequencies, means, and standard deviations, and presented in tables and text. The log‐binomial model is theoretically appropriate for estimating prevalence ratios [22]. However, it did not converge with our data. Therefore, we applied Poisson regression with robust variance, which is widely recommended as a reliable alternative for estimating prevalence ratios in cross‐sectional studies. Poisson regression with robust variance was used to identify factors associated with knowledge of PMTCT, and results were presented as prevalence ratios (PRs) with 95% confidence intervals (CIs). This approach was chosen because odds ratios derived from logistic regression in cross‐sectional studies can substantially overestimate relative risk when the outcome is common [23, 24]. Bivariate Poisson regression analysis with robust variance was first conducted to identify candidate variables. Those with a *p* value < 0.25 were included in the multivariable Poisson regression model with robust variance to control for potential confounders. This threshold was chosen based on methodological recommendations, which suggest that a more liberal cutoff at the bivariable stage helps avoid excluding potentially important predictors that may become significant in the multivariable model [25]. In the multivariable model, variables with a *p* value < 0.05 were considered statistically significant. Adjusted prevalence ratios (APRs) with 95% CIs were calculated to assess the presence, strength, and direction of associations between the dependent and independent variables.

#### 2.5.1 Missing Data Handling

All cases were complete, and therefore no imputation or exclusion procedures were required.

#### 2.5.2 Model Diagnostics of Modified Poisson Regression

To evaluate the associations between explanatory variables and knowledge of PMTCT, we employed modified Poisson regression with robust variance to directly estimate prevalence ratios. Model adequacy was evaluated using Pearson *χ* ^2^ and deviance statistics. Goodness‐of‐fit tests indicated no evidence of poor fit (deviance = 414.94, *p* = 1.000; Pearson *χ* ^2 ^= 457.25, *p* = 1.000), supporting the appropriateness of the model. Multicollinearity was assessed using variance inflation factors (VIF), with values ranging from 1.03 to 1.97, confirming the absence of problematic correlations among independent variables and the stability of regression estimates.

### 2.6 Ethics Approval and Consent to Participate

Ethical clearance was obtained from the Institute of Public Health, College of Medicine and Health Sciences, University of Gondar Ethical Review Committee (Ref. No: IPH/2107/2014 E.C). In addition, a support letter was obtained from the town administration office and shared with the kebeles. Participants received a clear explanation of the study objectives and provided written informed consent. They were informed that participation was voluntary and that they could withdraw at any time. All information collected was kept confidential, securely stored, and used exclusively for this study. The research was conducted in accordance with the principles of the Declaration of Helsinki.

## 3 Results

### 3.1 Socio‐Demographic Variable

Of the 816 pregnant women recruited, 803 were provided with complete information, with a response rate of 98%. The mean age of the pregnant women was 29.0 with SD ± 4.58, and 291(36.2%) pregnant women were between the age group of 30–34. More than half of the respondents (51.6%) were rural residents, and nearly one‐third of the study participants attended primary schools (Table 1).

**Table 1:** Socio‐demographic characteristics of the study participants at Debre Birhan, Ethiopia.

Variable	Category	Frequency	Percent (%)
Age	15–19	8	1.00
	20–24	144	17.9
	25–29	240	29.9
	30–34	291	36.3
	35–39	120	14.9
Marital status	Single	15	1.9
	Married	742	92.3
	Divorced	31	3.9
	Widowed	15	1.9
Religion	Orthodox	651	81.1
	Muslim	98	12.2
	Protestant	29	3.6
	Catholic	25	3.1
Residence	Urban	389	48.4
	Rural	414	51.6
Educational status	No formal education	123	15.3
	Primary education (1–8)	244	30.4
	Secondary education and above	436	54.3
Current occupation	House wife	394	49.3
	NGO	21	2.6
	Government employee	200	24.9
	Market vendor/trader	103	12.8
	Daily laborer	14	1.7
	Unemployed	33	4.2
	Private sector	38	4.5
Wealth index	Poorest	163	20.3
	Poorer	162	20.1
	Middle	157	19.6
	Richer	161	20.6
	Richest	160	19.4
Media exposure	Yes	667	83.1
	No	136	16.9
Average time to reach Health facility	< 30 min	624	77.7
	≥ 30 min	179	22.3

Abbreviation: NGO, non‐governmental organizations.

### 3.2 Reproductive‐Related Characteristics

Of the total pregnant women surveyed, 261 (32.5%) were primiparous, and 326 (40.6%) were multiparous. More than half of the participants, 445 (55.4%), were beyond 25 weeks of gestation at the time of interview. A majority, 640 (79.7%), reported current antenatal care (ANC) follow‐up, and 547 (68.1%) had a history of institutional delivery. These reproductive characteristics highlight that most participants were engaged with maternal health services, which has implications for their exposure to PMTCT information.

### 3.3 Information‐Related Characteristics

Of the total participants, 667 (83.1%) reported exposure to mass media, and 580 (72.2%) received health information about HIV/AIDS from healthcare providers. Approximately 316 (39.4%) pregnant women discussed HIV/AIDS and mother‐to‐child transmission with their husbands, while 363 (45.2%) reported discussing antenatal care with their husbands. In addition, 636 (79.2%) of the women had been tested for HIV. These findings suggest that while most participants were exposed to health information through media and providers, fewer engaged in spousal communication, which may influence knowledge uptake and decision‐making regarding PMTCT.

### 3.4 Knowledge of Women on Prevention of Mother‐to‐Child Transmission (PMTCT)

This study revealed that 288 (35.9%, 95% CI: 32.6–39.3) of the participants demonstrated good knowledge of PMTCT of HIV. A majority, 574 (71.5%), were aware that HIV can be transmitted from mother to child, while 229 (28.5%) did not know this mode of transmission. More than half, 508 (63.3%), correctly identified the timing of HIV transmission during pregnancy, delivery, or breastfeeding. However, only 326 (40.6%) of pregnant women reported knowledge of specific prevention strategies for mother‐to‐child transmission. These findings highlight substantial gaps in awareness, particularly regarding preventive measures, which may limit the effectiveness of PMTCT interventions (Table 2).

**Table 2:** Knowledge of PMTCT among pregnant women at Debre Birhan, Ethiopia.

Variable	Category	Frequency	Percent (%)
Know ways of preventing someone from acquiring HIV	Yes	417	51.9
	No	386	48.1
Know the mode of HIV transmission	Yes	318	39.6
	No	485	60.4
Know the mother‐to‐child transmission of HIV	Yes	574	71.5
	No	229	28.5
Know the time of HIV transmission from mother to child	Yes	508	63.3
	No	295	36.7
Know the prevention of mother‐to‐child transmission of HIV	Yes	326	40.6
	No	477	59.4
Know the ways to prevent HIV transmission from an infected woman to her baby	Yes	241	30.1
	No	562	69.9
Know preferable mode of delivery to prevent PMTCT	Yes	73	9.1
	No	730	90.9
Know the time of initiation of prophylaxis for women living with HIV	Yes	93	11.6
	No	710	88.4
Know the time to start ART prophylaxis for newborn	Yes	82	10.2
	No	721	89.8
Know about the increased risk of an infant acquiring HIV infection during breastfeeding	Yes	109	13.6
	No	694	86.4

### 3.5 Factors Associated With Knowledge of PMTCT of HIV

In the bivariate Poisson regression analysis with robust variance, several variables were associated with knowledge of PMTCT. Those included residence, wealth index, educational status, occupational status, current ANC, mother's discussion with husband about ANC, husband's discussion of HIV/AIDS and PMTCT, health information received from health care providers about HIV/AIDS and PMTCT, average time to reach a health facility, exposure to mass media, and gestational age. To minimize the effect of confounding factors, variables with a *p* value < 0.25 in the bivariate Poisson regression analysis were included in the multivariable model. In the final multivariable Poisson regression analysis with robust variance, four variables, residence, educational status, occupation, and receiving health information from healthcare providers about HIV and PMTCT, were independently associated with PMTCT knowledge.

Participants living in urban areas were 1.52 times (aPR = 1.52, 95% CI 1.22–3.19) more likely to have better knowledge of PMTCT for HIV than rural residents. Women who attended secondary education and above were significantly more likely to have better knowledge of PMTCT compared to women with no formal education (aPR = 1.94, 95% CI 1.23–3.09). Women employed in government organizations were significantly more likely to have better knowledge of PMTCT compared to housewives (aPR = 1.71, 95% CI 1.27–2.31). Women who were employed as market traders were 45% higher prevalence of knowledge compared to housewives (aPR = 1.45, 95% CI 1.03–2.03). Employment in private organizations was independently associated with higher PMTCT knowledge, with women in these jobs having a 58% higher prevalence of knowledge compared to housewives (aPR = 1.58, 95% CI 1.04–2.38). Women who received health information from their healthcare providers about PMTCT and HIV/AIDS were significantly more knowledgeable about PMTCT compared to women who did not receive such information (aPR = 1.60, 95% CI 1.14–2.26) (Table 3).

**Table 3:** Factors associated with knowledge of PMTCT among pregnant women at Debre Birhan, Ethiopia.

Variable	Knowledge of PMTCT		CPR [95% CI]	APR [95% CI]	*p* value
	Good knowledge	Poor knowledge			
Residence					
Urban	203	186	2.48 (2.05–3.14)	1.52 (1.22–3.19)**	0.000
Rural	85	329	1	1	
Educational status					
No formal education	19	104	1	1	
Primary education	27	217	1.72 (0.41–1.23)	0.76 (0.44–1.31)	0.329
Secondary and above	242	194	3.67 (2.35–5.48)	1.94 (1.23–3.09)**	0.005
Occupation					
Housewife	60	334	3.13 (1.88–5.19)	1.37 (0.81–2.33)	
NGO	10	11	4.50 (3.49–5.78)	1.71 (1.27–2.31)**	0.241
Government employee	137	63	2.87 (2.08–3.95)	1.45 (1.03–2.03)*	0.000
Market trade vendor	45	58	3.28 (1.85–5.83)	2.01 (1.12–3.60)*	0.031
Daily laborer	7	7	1	1	0.019
Unemployed	12	21	2.38 (1.43–3.97)	1.05 (0.94–2.42)	0.089
Private	17	21	2.94 (1.92–4.49)	1.58 (1.04–2.38)*	0.029
Average time to reach a health facility					
< 30 min	42	137	1	1	
≥ 30 min	246	378	0.59 (0.44–0.78)	0.95 (0.72–1.26)	0.742
Wealth index					
Poorest	26	137	1	1	
Poorer	32	130	1.24 (0.77–1.98)	1.07 (0.72–1.59)	0.714
Middle	64	93	2.56 (1.71–3.81)	1.25 (0.83–1.73)	0.325
Richer	75	86	2.92 (1.97–4.31)	1.21 (0.84–1.74)	0.298
Richest	91	69	3.56 (2.44–5.20)	1.36 (0.95–1.94)	0.089
Gestational age					
< 25 weeks	109	249	1	1	
≥ 25 weeks	179	266	1.32 (1.08–1.60)	1.1 (0.93–1.30)	0.243
Current ANC					
Yes	221	419	0.84 (0.68–1.03)	0.79 (0.64–0.97)	0.029
no	67	96	1	1	
Receiving health information from health care providers about HIV and MTCT					
Yes	257	323	3.19 (2.27–4.48)	1.6 (1.14–2.26)**	0.006
No	31	192	1	1	
Discussion with husband about MTCT and HIV/AIDS					
Yes	173	143	2.32 (1.92–2.78)	1.11 (0.88–1.38)	0.372
No	115	372	1	1	
Discussion with husband about ANC					
Yes	189	174	2.31 (1.89–2.83)	1.15 (0.92–1.43)	0.215
No	99	341	1	1	
Exposure to mass media					
Yes	260	407	1.89 (1.34–2.66)	1.03 (0.76–1.40)	0.807
No	28	108	1	1	

Abbreviations: APR, adjusted prevalence rate; CPR, crude prevalence rate.

*p‐value < 0.05

**p‐value <0.01.

## 4 Discussion

In this study, over one‐third (35.8%) of pregnant women demonstrated good knowledge of PMTCT. This level of awareness highlights persistent gaps in maternal health communication and suggests that many women remain vulnerable to misinformation or an incomplete understanding of HIV prevention strategies. The finding underscores the need for intensified community‐based education efforts, as facility‐based counseling alone may not reach women with limited access to the health system in the community. This finding aligns with results from Ethiopia (34.9%) [20], Southeast Nigeria (38.2%) [14], and Cameroon 37.0%, suggesting a regional pattern of moderate awareness [26]. However, our result is higher than those reported in Mecha District, Amhara region (22.4%) [18], Assosa (17.4%) [12], Meket District, Amhara [19, 27], South Sudan (30.7%) [28], and Pakistan (6%) [29]. The possible difference might be educational attainment. In Pakistan, three‐fourths of participants had less than primary education, whereas most women in our study had education beyond primary school. Higher educational status enhances comprehension of counseling messages, increases exposure to diverse information sources, and reduces susceptibility to myths, thereby strengthening PMTCT knowledge. This justification is heavily supported by existing evidence demonstrating that a woman's educational advancement beyond the primary level is significantly associated with higher PMTCT awareness [13, 16]. These findings highlight the role of female education in tailoring maternal health literacy and underscore the need to integrate health education into broader educational and community programs.

Conversely, our findings are lower than those reported in East Gojjam (52%) [13], Western Wollega 94.1% [30], Nigeria 65% [31], South Africa (87%) [32], Tanzania (46%) [33], Vietnam (46.83%) [34], and India (50.8%) [35]. The possible explanation might be the study setting and target population characteristics. Facility‐based studies often report higher knowledge because women recruited during ANC visits are regularly exposed to provider counseling and health education. In contrast, community‐based designs, such as the one employed in our study, capture a broader demographic, including women with minimal health system contact, thereby reducing their opportunities for immediate counseling and clinical information exchange. Study populations also matter: for example, the Western Wollega study focused on HIV‐positive women, who typically receive more intensive counseling and follow‐up care, resulting in higher awareness. Evidence from Tanzania further supports this, showing that HIV‐positive women have better knowledge of PMTCT than those who are not infected [36].

Women living in urban areas were more likely to have better knowledge of PMTCT compared to rural residents. This disparity might be explained by differences in access to information and services. Urban women benefit from greater exposure to diverse communication channels, including mass media, the Internet, and social networks, which facilitate the dissemination of health messages. They might have better access to schools and health facilities, which enhances literacy and increases opportunities for counseling. In contrast, rural women might face barriers such as limited media coverage, weaker educational infrastructure, and reduced contact with healthcare providers, leaving them more vulnerable to misinformation. These findings underscore the need for targeted community‐based interventions in rural areas, including strengthening health outreach programs and expanding access to reliable information sources.

Educational attainment was also associated with PMTCT knowledge. Women with secondary education and above were more knowledgeable about PMTCT compared to those with no formal education. The possible reason might be that secondary schooling provides exposure to sexual and reproductive health information and enhances literacy, which facilitates comprehension of counseling messages. Evidence revealed that educated women have higher odds of understanding and adopting health messages, while uneducated women remain vulnerable to misconceptions [33, 34]. These findings implicate the importance of expanding educational opportunities for girls and integrating health literacy into school curricula and community programs as long‐term strategies to strengthen PMTCT awareness.

Women employed by the government demonstrated better knowledge of PMTCT compared to housewives. This association may reflect greater opportunities for social networking within the workplace, which facilitates the exchange of health information among colleagues [13]. It may also be linked to higher educational attainment, socioeconomic stability, and exposure to workplace health programs, factors that have been associated with improved awareness of maternal and child health issues. These findings suggest that women outside formal employment, particularly housewives, may require targeted community‐based education strategies to ensure equitable access to PMTCT information.

Women who are market trade vendors are more likely to have better knowledge of PMTCT compared with housewives. This may be explained by the frequent social interaction vendors have with customers, peers, and community networks, which increases their opportunities to receive and exchange health‐related messages.

Women engaged in daily labor are more likely to have better knowledge of PMTCT compared with housewives. The possible explanation might be due to differences in social exposure and access to health information. Daily laborers often work in environments where they interact with diverse groups of people, which increases their likelihood of encountering health messages through peers, community programs, or workplace initiatives [30]. This occupational engagement may also foster greater independence and mobility, enabling them to access health facilities and educational campaigns more frequently.

Women working in private companies are more likely to have better knowledge of PMTCT compared with housewives. This might be linked to differences in education, socioeconomic status, and workplace exposure. Women working in formal employment settings often have higher levels of education and literacy, which enhances their ability to understand and retain health‐related information. In addition, private companies may provide health awareness programs, insurance benefits, or workplace campaigns that expose employees to maternal and child health topics, including PMTCT.

Women who received health information about HIV and PMTCT from healthcare providers were more knowledgeable than those who did not. Healthcare professionals are among the most trusted and authoritative sources of health information for pregnant women [37, 38]. A possible explanation is that healthcare providers are uniquely positioned to deliver accurate, evidence‐based messages, reducing women's vulnerability to myths and misconceptions about HIV/AIDS that circulate within communities [38, 39]. Women who receive counseling and information directly from providers are more likely to understand PMTCT strategies and adopt preventive practices.

### 4.1 Strength and Weakness

This study has several strengths, including the use of a validated tool with acceptable reliability, a large and representative sample, and the application of robust statistical methods (Poisson regression with robust variance) that allowed direct estimation of prevalence ratios. Nonetheless, several limitations must be acknowledged. Reliance on self‐reported data introduces the possibility that information bias, interviewer bias, social desirability, and memory biases may have influenced responses. The exclusion of unregistered pregnancies limits external validity and may underestimate knowledge gaps among marginalized women. Finally, the absence of a qualitative component limits contextual understanding of barriers, cultural perceptions, and women's lived experiences, which could enrich the interpretation of quantitative findings. Despite these limitations, the findings have important programmatic implications: strengthening antenatal care education, expanding outreach to women outside the formal health system, and incorporating qualitative approaches in future research to capture contextual barriers and women's perspectives could enhance PMTCT knowledge and uptake.

## 5 Conclusion

This study found that only 35.9% of pregnant women had good knowledge of PMTCT, highlighting a substantial gap in awareness. Addressing this gap requires targeted interventions that strengthen provider‐patient counseling, actively involve partners in educational sessions, and expand outreach to women without ANC follow‐up, particularly in rural and socioeconomically disadvantaged settings. These findings have direct policy and programmatic implications for maternal and child health services, as improving PMTCT knowledge contributes to reducing HIV transmission and advancing the Sustainable Development Goals, notably SDG 3 (ensuring healthy lives and promoting well‐being for all) and SDG 5 (achieving gender equality and empowering all women and girls).

## Author Contributions

**Lewegneh Wegayehu Tessema:** conceptualization, methodology, formal analysis, validation, software, data curation, writing – review and editing, writing – original draft. **Yazachew Moges Chekol:** conceptualization, validation, software, data curation, writing – original draft. **Elsa Awoke Fentie:** methodology, validation, supervision, writing – review and editing. **Abebaw Addis Gelagay:** methodology, validation, data curation, supervision, writing – review and editing.

## Funding

This study received no specific funding for this study.

## Conflicts of Interest

The authors declare no conflicts of interest.

## Transparency Statement

The lead author, Lewegneh Wegayehu Tessema, declares that this manuscript is a true, accurate, and transparent account of the study being reported; that no important aspects of the study have been omitted. Any discrepancies from the study as planned have been explained.

## Data Availability

The data that support the findings of this study are available on request from the corresponding author. The data are not publicly available due to privacy or ethical restrictions.
